# Problem-based learning in obstetrics and gynecology: a systematic review

**DOI:** 10.61622/rbgo/2025rbgo68

**Published:** 2025-12-09

**Authors:** Ivan Fernandes, Cibele Isaac Saad Rodrigues, Henri Augusto Korkes, Luiz Ferraz de Sampaio

**Affiliations:** 1 Pontifícia Universidade Católica de São Paulo Faculdade de Ciências Médicas e da Saúde Sorocaba SP Brazil Faculdade de Ciências Médicas e da Saúde, Pontifícia Universidade Católica de São Paulo, Sorocaba, SP, Brazil.

**Keywords:** Problem-based learning, Gynecology, Obstetrics, Education, medical

## Abstract

**Objective:**

Evaluate the application and effectiveness of the problem-based learning (PBL) teaching model in Obstetrics and Gynecology (OB/GYN) undergraduate education.

**Data sources:**

A systematic search was performed in five electronic databases (LILACS, PubMed/MEDLINE, EMBASE, CINAHL, and Cochrane Library) for studies published between January 1, 2000, and December 31, 2021. The search strategy used descriptors in English, portuguese, and Spanish based on the PICO framework: "Education, Medical, Undergraduate" (P); "Problem-Based Learning", "Gynecology", and "Obstetrics" (I); no comparator (C); and "Patient Satisfaction" or "Knowledge" (O). Study selection: A total of 24 studies were included. Selection criteria encompassed original articles that evaluated the impact of PBL in OB/GYN undergraduate education, without language restrictions.

**Data collection:**

Two independent reviewers extracted data regarding study characteristics, participant profiles, methodology, and outcomes. Discrepancies were resolved by consensus.

**Data synthesis:**

Among the included studies,14(58.33) of them coming from Asian, most of them 16(66.66) having been published between the years 2006-2010 and 2016-2021. About 15(62.5) dealt only with students, the majority consisting of surveys/questionnaires 8(30) and non-randomized comparative studies 7(29.16). As for academic performance, 14(58.33) demonstrated that the PBL methodology is superior considering the ability to sediment information, and 8(30) indicated PBL is superior concerning developing clinical reasoning. Regarding student satisfaction, 11(45.83) of the studies indicated a positive experience with the method.

**Conclusion:**

The PBL model appears more effective than traditional teaching methods in enhancing clinical competence and student satisfaction in OB/GYN education. However, further well-designed studies are necessary to confirm these findings and guide educational strategies.

**PROSPERO Registry:**

CRD42021282337

## Introduction

When trying to overcome potential gaps in knowledge and performance of clinical skills, as well as to constantly improve the acquisition of new knowledge in healthcare areas, it is critical to physicians graduated in the last few years to make an effort to "learn to learn" and thus stay informed and updated. With this objective, some pedagogical proposals aim to offer new strategies to review classic concepts on how to teach in medical schools in the last decades.^(1,2)^

Several methods that fit into the concepts of modern and critical pedagogical philosophy can be used in academic settings. Their characteristics may be differentiated between cooperative and collaborative techniques. In the context of medical education, problem-based learning (PBL) gains traction.^(2,3)^

Since its elaboration and pioneering use in medical education in the McMaster University, Canada, in the late 1960s, the pedagogical approach involves issues described in scenarios that allow students to identify their own learning goals. Overall, these situations are patient-centered, and the learning creation dynamics aims to develop skills simultaneously related to problem resolution and the establishment of scientific and clinical knowledge bases.^(3)^

In general, PBL sessions consist of small groups with about eight to ten students and one facilitating professor, positioned to aid in the process. Through two sessions, students discuss scenarios ruled by the Maastricht seven steps, whose structure may vary between institutions. In the first session, the group chooses one student to act as secretary of the meeting and another to be the coordinator. Both will assume functions to conduct the session and register the thinking process of the group, respectively.^(4)^

In the second session, participants discuss the results of each student's individual studies, enabling the exchange of information and ideas. Every participant can question and share their knowledge, therefore teaching their colleagues. The facilitating professor oversees the whole process, ensuring that students stay on topic during sessions and providing guidance, when needed.^(4)^

Following the first PBL tutorial, each student uses his own resources and strategies to reach the knowledge required as identified by the PBL session, prior to the next PBL group meeting.^(4)^

Regarding the application of concepts of active methodologies, especially PBL, when teaching medical specialties, Obstetrics and Gynecology (OB-GYN) gains prominence. Several studies in the literature, including Nalesnik et al. (2004),^(4)^ Casey et al. (2005)^(5)^ and Bi et al. (2021),^(6)^ demonstrate that the structured steps in PBL were associated to higher student and faculty satisfaction and did not have a negative impact in students’ grades.^(4-6)^

From the 1980s onwards, driven by the universal observation of obsolescence in traditional teaching practices in medical schools and faced with the new needs that emerged at the end of the 20th century, official movements emerged to update the medical teaching proposal, including at our university. Due to the need to adapt to innovative concepts in medical education, the institution adopted a new pedagogical strategy, with a project presented to the academic community, approved and implemented since 2006, in which active teaching and learning methodologies gain priority, including ABP. In this context, according to the Pedagogical Project of the Medicine Course (PPC), teaching in GO is included regularly, starting in the third year with the initial module. However, it returns in the 4th, 5th, and 6th year stages, which corresponds to the medical internship in this model.^(7)^

The increasing use of PBL in the last years has created a unique problem. Oftentimes, facilitators support their teaching methods in their own past learning experiences and, considering how relatively recent PBL is, faculty usually is not familiar with the process and dynamics of this form of active methodology.^(3,6)^ Therefore, the objective of this study is to evaluate the role of PBL in OB-GYN education using a systematic review.

## Methods

This study is a systematic review, descriptive in nature, structured based on the methodology proposed by Tranfield et al.,^(8)^ whose article titled "Towards a Methodology for Developing Evidence-Informed Management Knowledge by Means of Systematic Review" was published on the British Journal of Management in 2003.^(8)^

This systematic review was registered on the International Registry of Prospective Systematic Reviews (PROSPERO), available at [https://www.crd.york.ac.uk/prospero/display_record.php?RecordID=282337].

The entire investigation process was organized in three steps: planning (step I), conduction (step II) and reporting and dissemination (step III) of the review.

First, we have elaborated the investigation question that the systematic review intended to answer. The primary investigation question was "What is the efficacy of PBL methodology when teaching Obstetrics and Gynecology to medical students?" And the secondary question was "What is the perception and satisfaction of medical students regarding the use of PBL in Obstetrics and Gynecology education?"

To identify meaningful studies, we have performed searches in 5 electronic databases indexed with the following MeSH terms in English and their respective correlates in Portuguese and Spanish: Problem-Based Learning; Education, Medical; Gynecology; Obstetrics; Education, Medical, Undergraduate; Patient Satisfaction; Knowledge. The PICO strategy (acronym for P: population/patients; I: intervention; C: comparison/control; O: outcome) was used according to the research question linked to each subject area. A bibliographic survey was performed in the 5 databases currently available for medical literature: LILACS, PubMed/MEDLINE, EMBASE, CINAHL and Cochrane between January 1, 2010 and December 31, 2021.

The bibliographic survey and the selection of titles, abstracts and full texts were independently performed, in accordance with the investigation protocol described in phase I (Identifying the need for a systematic review, preparing a systematic review proposal and developing a systematic review protocol), by three main reviewers (the authors). Inclusion and exclusion criteria were applied as follows:

Inclusion criteria:

- Publications with national and international relevance indexed in scientific databases; classified in the highest strata of the Brazilian QUALIS grading system;
- PBL methodology was the main subject of the research;
- Publications solely related to teaching experiences of Obstetrics and Gynecology concepts to medical students and studies compliant with controlled index terms defined to each area.

Exclusion criteria:

- Studies including other types of participants, such as postgraduate medical students, residents, and students of other non-medical specialties;
- Every publication not including PBL methodology and duplicate content;
- Letters to the editor, editorials, books and theses/dissertations;
- Publications graded lower than C in the Brazilian QUALIS grading system.

Studies that have received at least two out of three positive evaluations were selected for the next step. Extraction and interpretation of significant data was carried out through more in-depth reading, with constant monitoring of the completion of all steps. This process was also under oversight by three reviewers.

This review resulted in the inclusion of 82 publications, including three scientific texts in grey literature. Of those, 21 studies were excluded due to duplicity and 21 were removed after abstract evaluation for not complying with the inclusion criteria, either with the PICO strategy used or because they presented methodological errors. The scientific texts found in grey literature were excluded since all of them were textbooks. In the end, 24 studies were selected for data extraction and qualitative synthesis.

Data extracted from selected studies were categorized and correlated in the results section in the following areas: characteristics of the selected publications and results evaluated (Appendices 1 and 2).

## Results

The study design according to main and secondary outcomes and continents is shown in chart 1 for better comprehension. It should be noted that studies have different designs. Therefore, the results presented by specific articles do not necessarily imply contrary results for other studies.

**Chart 1:** Primary and secondary results and continents of origin of the studies

		Survey or questionnaire	Non-randomized comparative study	Randomized comparative study	Descriptive experience	Systematic review and meta-analysis
Number of studies		7	6	6	3	2
Results of academic performance						
	Consolidation of knowledge (n=13)	3	5	4	-	1
	Performance in academic exams (n=7)	-	4	2	1	-
	Development of clinical reasoning (n=9)	3	1	4	1	-
	Development of clinical competencies (n=6)	1	-	4	-	1
	Development of self-learning (n=4)	2	1	1	-	-
Results of teaching-learning process						
	Student satisfaction (n=9)	5	-	2	1	1
	Professor satisfaction (n=5)	4	1	-	-	-
Results of development of other competencies						
	Development of teamwork (n=2)	1	-	1	-	-
	Social and/or communication competencies (n=2)	-	-	1	1	-
Continents						
	Asia	2	3	6	1	1
	North America	3	2	-	2	-
	South America	1	1	-	-	1
	Europe	1	-	-	-	-

Regarding the countries of origin of the selected studies, 11(45.8) were from China, 6 (25.0) from the United States, 2(8.3) from Chile, 1(4.1) from India, 1(4.1) from Pakistan, 1(4.1) from Canada, 1(4.1) from Brazil, and 1(4.1) from Denmark. Regarding the continents of origin of the selected studies, 13(54,1) were from Asia, 7 (29.1) from North America, 3(12.5) from South America and merely 1(4.1) from Europe.

**Figure 1:**
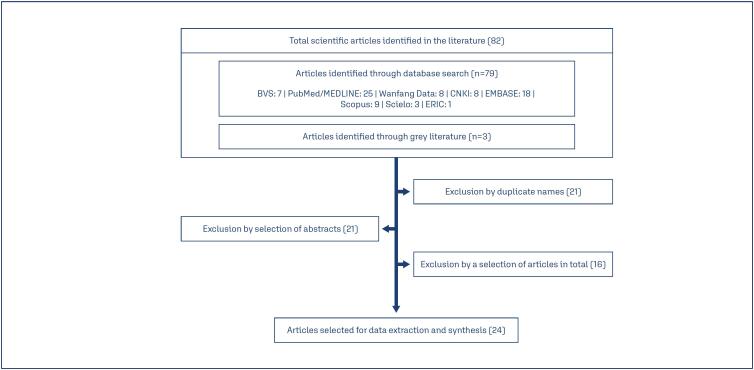
PRISMA Flow Diagram of the study design

The results of this item can be seen in chart 2.

**Chart 2:** Selected studies by year of publication

	n(%)
2000 - 2005	5(20,83)
2006 - 2010	8(33,33)
2011 - 2015	5(20,83)
2016 - 2021	6(25,00)
2000 - 2021	24 (100)

Concerning the characteristics of the sample, 15(62.5) of the studies have worked with students, 7(29.1) with students and professors and 2(7.69) did not specify the sample.

About the study design, 7(29.16) of the studies were classified as surveys or questionnaires, 6(25) were categorized as non-randomized comparative study, 6(25) as randomized comparative study, 3(12.5) as descriptive experience and 2(8.33) as systematic review and meta-analysis.

Comparisons presented in the studies, 12(50) implemented comparisons with conventional education, 11(45.83) did not perform clear comparisons and 1(8.33) made comparisons with conventional PBL.

In the selected sample, 13(54.16) have demonstrated that PBL has a superior ability to consolidate information. Of these, 3(23.07) of these studies are surveys or questionnaires, 4(30.76) are randomized comparative studies, 5(38.46) are non-randomized comparative studies and 1(7.69) are systematic reviews and meta-analyses. Furthermore, 3(23.07) of the studies had students and professors as study participants and 10(76.92) had only students. Out of these 13 studies in which PBL superiority was determined, 9(69.23) used conventional teaching as a comparison group and 4(30.76) did not have a specific comparison group.

Furthermore, 7(30.7) of studies assessed the performance in evaluation tests as an outcome. Of these, 5(71.42) show that students who went through PBL structured steps had a superior performance, 1(14.28) do not show differences between PBL and comparison groups and 1(14.28) show that PBL was inferior to the comparison group in this regard. It is possible to describe that 4(57.14) of these studies are non-randomized comparative studies, 2(28.57) are randomized comparative studies and 1(14,28) are descriptive experiences. Furthermore, 1(14.28) of the studies had students and professors as study participants and 6(85.71) had only students. Out of these 7 studies in which academic performance was assessed, 3(42.85) used conventional teaching as a comparison group, 3(42.85) did not have a specific comparison group and 1(14.28) had conventional PBL as control group (in this case, PBL was evaluated with some institutional modifications).

In 9(33.33) of all investigations, it was found that students who underwent PBL's organized processes had improved clinical reasoning. Out of these studies, 1(11.11) are non-randomized comparative studies, 4(44.44) are randomized comparative studies, 3(33.33) are surveys or questionnaires and 1(11.11) are descriptive experiences. Also, 3(33.33) of these studies had students and professors as study participants and 6(66.67) had only students. Finally, 5(55.55) of these studies used conventional teaching as a comparison group and 4(44.44) did not have a specific comparison group.

Furthermore, 6(25.00) of the studies examined clinical skill application as an outcome. Out of these studies, 5(83.33) have demonstrated that PBL made a greater contribution to the development of clinical skills than the article's comparison group while 1(16.6) were not able to demonstrate significant differences in this outcome. There are 4(66,66) randomized comparative studies, 1(16.6) systematic reviews and meta-analyses, and 1(16.6) surveys or questionnaires. Also, 1(16.6) of these studies had students and professors as study participants and 5(83.33) had only students. Finally, 5(83.33) of these studies used conventional teaching as a comparison group and 1(16.6) did not have a specific comparison group.

Furthermore, 4(16.66) of all research discovered that students who went through PBL planned steps enhanced their self-learning ability. It is worth noting that 1(25) of these studies are non-randomized comparative studies, 1(25) are randomized comparative studies and 2(50) are surveys or questionnaires. Also, 1(25) of these studies had students and professors as study participants and 3(75) had only students. Finally, 2(50) of these studies used conventional teaching as a comparison group and 2(50) did not have a specific comparison group.

Regarding student satisfaction, 9(37.5) of the studies have shown that the experience was deemed as positive by students who went through PBL structured steps. There are 5(55.55) surveys or questionnaires, 2(22.22) randomized comparative studies, 1(11.11) descriptive experiences, and 1(11.11) systematic reviews and meta-analyses. Also, 4(44.44) of these studies had students and professors as study participants, 4(44.44) had only students, and 1(1.11) did not specify the sample. Out of these 9 studies in which PBL was approved by the students, 4(44.44) used conventional teaching as a comparison group, 4(44.44) did not have a specific comparison group and 1(11.11) had conventional PBL as control group (in this case, PBL was evaluated with some institutional modifications).

Considering faculty satisfaction, 5(20.83) of the studies have shown that the experience was deemed as positive by professors who have applied PBL structured steps. It is worth mentioning that surveys or questionnaires account for 4(80) of these investigations, whereas non-randomized comparison studies account for 1(20). Also, 4(80) of these studies had students and professors as study participants and 1(20) had only students. Finally, 2(40) of these studies used conventional teaching as a comparison group and 3(60) did not have a specific comparison group.

Regarding other skills, 4(16.66) of the studies have shown that PBL structured steps granted students the ability to develop other skills. Out of these studies, 2(50) indicate that PBL has generated better development of teamwork and 2(50) indicate that PBL has contributed to the communication skills of students. Notably, 2(50) of these studies are randomized comparison studies, 1(25) are surveys or questionnaires, and 1(25) are descriptive experiences. Furthermore, all of these studies 4(100) used only students as study subjects. Finally, 2(50) of these studies used conventional teaching as a comparison group and 2(50) did not have a specific comparison group.

## Discussion

It is possible to highlight that, among places of origin of the selected studies, Asian countries seem to be more interested in the study of PBL applied to OB-GYN education, followed by countries in North America, South America, and Europe. This is similar to the result obtained when PBL is applied to medical education in general, according to a scoping review by Trullàs et al.^(9)^ Although PBL is applied in basically every continent, this trend reveals that there is more experience in its use in Asian and North American countries, and this is not different in OB-GYN education. Also, the most scientifically relevant studies are from Asia, especially China.^(6)^

Regarding the date of publication, most studies in our selection were carried out from 2006 and 2010 and from 2016 and 2021, also following a trend reported in the scoping review by Trullàs et al,^(9)^ which evaluated PBL applied to the teaching-learning process of medical content in general. Specifically in our service, we must highlight that PBL was implemented in 2006, at a time when this methodology was on the rise around the world.

The studies comprising our selection are highly heterogeneous. Most were performed with students as participants in various study designs, ranging from survey/questionnaire from systematic review and meta-analysis. In most studies, PBL was a compared to conventional education techniques. It is possible to note that this heterogeneity is also true when we assess PBL application in medical education in general, as in the scoping review by Trullàs et al.^(9)^ We can therefore state that the use of PBL in OB-GYN education follows the same pattern.

Even considering this heterogeneous character, the studies tend to show favorable results of the use of PBL in OB-GYN education regarding the academic performance of students, especially when compared to conventional education (expository). In several studies, we also note that students have felt safer about consolidation of information ^(5,6,10-20)^ and their performance in evaluation tests.^(12,19,21-23^^)^ Some studies report a greater contribution to the development of practical clinical skills^(6,11,16,18,20,24)^ development of clinical reasoning^(10,11,14-17,24-27)^ and self-learning ability^(14,15,26)^ despite the need for more studies to strengthen these findings. This is probably due to the exposure of small groups of students to a problem that must be solved. That situation makes them work together to find a solution and requires questioning, autonomy to study and development of communication among peers.^(28)^

Regarding student satisfaction with the use of PBL methodology in OB-GYN teaching-learning process, the studies have shown that most students reported being strongly satisfied, especially in comparison with conventional education techniques.^(4-6,11,13,14,20,29-31^^)^ It is assumed that the underlying reason for this outcome is the exposure to real-world cases and the encouragement of students to take a leading role in the construction of their knowledge, both typical characteristics of PBL methodology.^(32)^ Some studies also show greater faculty satisfaction.^(4,10,14,26,29)^ However, this outcome needs to be verified in further studies.

Although PBL applied to OB-GYN education seems to be effective, there are studies, even if they are a minority of our selection, which show divergent results regarding performance in evaluation tests.^(21,31)^ Furthermore, PBL has been criticized by some authors in literature. While some report that the experience in small PBL groups is inherently variable and sometimes dysfunctional, others indicate that self-directed learning is not appropriate since it is deemed as a self-education technique. There are also authors indicating that professors can be employed beyond the role of facilitator.^(28)^

On the other hand, based on the results of our study, we are able to state that PBL methodology in OB-GYN education is an interesting tool due to its potential benefits and its importance in the curriculum of medical schools, especially in our school (PUC-SP).^(5)^ Despite its limitations, also found in any other methodology, it is likely that PBL can make a greater contribution to the development of several other skills required in OB-GYN practice when compared to conventional education techniques.

Thus, we expect PBL methodology to evolve, adapting itself to ever-changing social and educational contexts, allowing its strengths to be maximized and its weaknesses to be addressed.

## Conclusion

Despite limitations subject to criticism in literature, PBL is a tool that has potential benefits in the study of OB-GYN in comparison with conventional methodologies. PBL may contribute to the development of skills required in the practice of these medical specialties, especially regarding academic performance and student satisfaction. Considering other potential outcomes, such as faculty satisfaction and development of clinical skills, additional studies are needed to evaluate these relationships.

## Data availability

: The authors did not make the data from this article available in repositories prior to submission.

## Appendix 1 Search strategy

**Table:**

PICO Element	Conceptual Description	MeSH Terms (English)
Population (P)	Medical students enrolled in undergraduate programs, particularly in Gynecology and Obstetrics.	Medical Student Medical Students Student, Medical Medical Education Education, Undergraduate Medical Medical Education, Undergraduate Undergraduate Medical Education Education, Graduate Medical Graduate Medical Education Medical Education, Graduate Gynecology Obstetrics
Intervention (I)	Use of Problem-Based Learning as an educational method.	Active Learning Curricula, Problem-Based Curriculum, Problem Based Curriculum, Problem-Based Experiential Learning Learning, Active Learning, Experiential Problem-Based Learning Problem Based Curriculum Vitae Problem Based Learning Problem-Based Curriculum Vitae Problem-Based Curriculum
Comparison (C)	Traditional teaching methods such as lectures or teacher -led instruction.	Lecture
Outcome (O)	Evaluation of student satisfaction, knowledge acquisition, and professional attitudes.	Patient Satisfaction Base, Knowledge Base, Knowledge (Computer) Bases, Knowledge Bases, Knowledge (Computer) Knowledge Base Knowledge Base (Computer) Knowledge Bases (Computer) Knowledgebase Knowledgebases

To retrieve articles that were not indexed in different databases, we used the corresponding descriptors in Spanish and Portuguese. We also included widely used terms for cataloging such articles; however, these are not classified as MeSH terms.

## Appendix 2 Synthesis of the studies

**Table:**

Authors	Title	Year	Country of origin	Periodical	Population (P)	Intervention (I)	Comparisons (C)	Outcomes (O)	Study design
Nalesnik, SW, Heaton, JO, Olsen, CH, Haffner, WH and Zahn, CM	Incorporating problem-based learning into an obstetrics / gynecology clerkship: Impact on student satisfaction and grades	2004	USA	American Journal of Obstetrics and Gynecology Vol. 190(5), pp. 1375-1381	Students/ Tutors	Learning Based on in Problems	Learning traditional	Body satisfaction teacher positive body satisfaction student positive	Search or questionnaire
Ping, LI, Yue-zhen, XUE, Yang, ZOU and Qing, MIAO	Application of transitional problem-based learning in teaching of Department of Gynecology and Obstetrics	2008	China	Journal of Shanghai Jiaotong University (Medical Science), pp. S1	Students/ Tutors	Learning Based on in Problems	Learning traditional	Body satisfaction teacher positive Greater participation student Greater student interest Greater reasoning clinical Greater knowledge retention	Study comparative no randomized
Sørensen, J.L., Tabor, A., Ottesen, B. and Aspegren, K.	Experiences with the implementation of problem-based learning (PBL) during medical students ‘ clinical training in obstetrics and gynecology [Erfaringer med implementing af problembaseret læring (PBL) under medicinstuderendes clinic ophold i gynækologi	2004	Denmark	Ugeskrift for Laeger Vol. 166(21), pp. 2005-2009	Students/ Tutors	Learning Based on in Problems	No comparison	Greater reading and greater reflection Greater usefulness of PBL	Search or questionnaire
Casey, P.M., Magrane, D. and Lesnick, TG	Improved performance and student satisfaction after implementation of a problem-based preclinical obstetrics and gynecology curriculum	2005	USA	American Journal of Obstetrics and Gynecology Vol. 193(5), pp. 1874-1878	Students	Learning Based on in Problems	Learning traditional	Greater knowledge retention Body satisfaction student positive	Search or questionnaire
Augusthy, V.	A comparative study of the learning outcomes and students ‘ satisfaction from problem-based learning and lecture-based learning in obstetrics and gynecology	2016	India	International Journal of Reproduction, Contraception, Obstetrics and Gynecology Vol. 5(5), pp. 1368-1374	Students	Learning Based on in Problems	Learning traditional	Greater reasoning clinical Increased problem - solving skills Increased knowledge retention Body satisfaction student positive	Study comparative and randomized
Ronco, R., Munoz, G. and Prado, P.	Problem-based learning in tiny tots and mothers-to-be	2007	Chile	Medical Education Vol. 41(5), pp. 512-512	Students	Learning Based on in Problems	No comparison	Greater reasoning clinical Greater reading and greater reflection Greater self-learning Body satisfaction teacher positive	Search or questionnaire
Tayyeb, R	Effectiveness of problem based learning as an instructional tool for acquisition of content knowledge and promotion of critical thinking among medical students	2013	Pakistan	Journal of the College of Physicians and Surgeons -- Pakistan: JCPSP Vol. 23(1), pp. 42-46	Students	Learning Based on in Problems	Learning traditional	Greater reasoning clinical Greater skill in the problem solving	Study comparative and randomized
Dunn, T. S., Wolf, D., Beuler, J. and Coddington, CC	Increasing recruitment of quality students to obstetrics and gynecology: impact of a structured clerkship	2004	USA	Obstetrics and gynecology Vol. 103(2), pp. 339-341	Students/ Tutors	Learning Based on in Problems	No comparison	Body satisfaction teacher positive body satisfaction student positive	Search or questionnaire
Pomerantz, T., Patel, P., Miller, K., Ziegler, C., Hoffmann, J. and Bergin, A.	Teaching Medical Students About Abortion Through Problem-Based Learning [1O]	2018	USA	Obstetrics & Gynecology Vol. 131(1), pp. 163S-163S	Students	Learning Based on in Problems	Previous status	Greater knowledge retention Lower performance in the assessment tests	Study comparative no randomized
Lobb, DK, Inman, DR and Butler, RG	Problem-based learning in reproduction physiology.	2004	Canada	Journal of midwifery & women's health Vol. 49(5), pp. 449-453	Students/ Tutors	Learning Based on in Problems	No comparison	No specified	Experience descriptive
Bi, S., Liu, R., Li, J. and Gu, J.	The effectiveness of problem-based learning in gynecology and obstetrics education in China: A meta-analysis of randomized controlled trials	2021	China	Medicine Vol. 100(9), pp. e24660	Students/ Tutors	Learning Based on in Problems	Learning traditional	Greater knowledge retention Body satisfaction student positive Majors practice skills clinic Highest score in operations clinics	Revision systematic and meta-analysis
Pomerantz, T., Bergin, A., Miller, K.H., Ziegler, CH. and Patel, P.D.	A Problem-Based Learning Session on Pregnancy Options, Counseling, and Abortion Care	2019	USA	MedEdPORTAL Vol. 15	Students	Learning Based on in Problems	Previous status	Greater knowledge retention Better performance in the assessment tests	Study comparative no randomized
Spencer, AL and McNeil, M.	Interdisciplinary curriculum to train internal medicine and obstetrics-gynecology outpatient residents women's health: adapting problem-based learning to residency education	2009	USA	Journal of women's health (2002) Vol. 18(9), pp. 1369-1375	Resident doctors	Learning Based on in Problems	Traditional PBL	Greater knowledge retention Body satisfaction student positive	Search or questionnaire
Ortega-Cortez, A., Espinoza-Navarro, O., Ortega, A. and Brito-Hernández, L	Performance of University Students en Signatures of the Morphological Sciences: Use of Active Based Learning in Problems (ABP)	2021	Chile	International Journal of Morphology Vol. 39(2), pp. 401-406	Students	Learning Based on in Problems	Learning traditional	Better performance in the assessment tests	Study comparative no randomized
Jin, JIN and ZHANG, G.-w.	Application of PBL+ LBL teaching methods to eight-year medical students in gynecology and obstetrics clinical teaching [J]	2011	China	Basic Medical Education Vol. 2	Students	Learning Based on in Problems	Traditional PBL	No difference in performance in the evaluation tests Body satisfaction student positive	Study comparative and randomized
CHENG, M., Jiangyan, LOU and Xin, TAN	Advantages and challenges of PBL teaching in obstetrics and gynecology	2009	China	China Higher Medical Education	Students/ Tutors	Learning Based on in Problems	No comparison	Greater knowledge retention Greater reasoning clinical Self-learning Body satisfaction student positive body satisfaction teacher positive	Search or questionnaire
Wenqi, T.	Application of "PBL" in Probation of Obstetrics and Gynecology	2006	China	Researches in Medical Education, pp. 5	Students	Learning Based on in Problems	Learning traditional	Greater knowledge retention Greater self - learning Greater knowledge retention Body satisfaction student positive	Study comparative no randomized
Yonghong, LUO and Lianzhi, H.E.	Application of CBL and PBL for clinical teaching of gynecology and obstetrics [J]	2013	China	Basic Medical Education Vol. 3	Students	Learning Based on in Problems	Learning traditional	Greater knowledge retention Majors practice skills clinic Greater reasoning clinical Major Work as a team	Study comparative and randomized
Jean-Baptiste, S.	Does a Problem Curriculum-Based Enhance Knowledge of CREOG Objectives in an Obstetrics and Gynecology Residency Program ?	2019	Russia	Obstetrics & Gynecology Vol. 134	Doctors Residents	Learning Based on in Problems	Learning traditional	No difference in performance in evaluative tests Body satisfaction student positive	Study comparative no randomized
LI Shu-qin, SUN Qing, HE Lianzhi	Application of problem-based learning approach to clinical practice of gynecology and obstetrics	2012	China	J of Wannan Medical College 2012; 31(3)	Students	Learning Based on in Problems	Learning traditional	Greater knowledge retention Greater reasoning clinical Greater communication skills Self - learning	Study comparative and randomized
Liu Chuan, Liu Caixia, Zhao Yan, Fang Yuanyuan, Guo Zhiqiang, Li Na	A preliminary study on the application of PBL model in the production practice teaching of obstetrics and gynecology	2008	China	China Higher Medical Education 2008 No.9	Students	Learning Based on in Problems	Learning traditional	No differences us skills development clinics Greater knowledge retention Better performance in the assessment tests	Study comparative and randomized
SUN Qing, HE Lianzhi, KONG Lina, NI Guantai	Experience and reformation of clinical clerkship teaching in obstetrics and gynecology	2011	China	CHINA MODERN MEDICINE	Students	Learning Based on in Problems	Learning traditional	Best skills clinics Greater knowledge retention Better performance in the assessment tests	Study comparative no randomized
Yang Huaguang Li Yi	Experience of applying PBL teaching clinical model teaching of advanced students in obstetrics and gynecology	2010	China	Journal of Shanxi Medical University: Basic Medical Education Edition	Students	Learning Based on in Problems	No comparison	Best skills clinics Greater knowledge retention Body satisfaction student positive Better work as a team	Search or questionnaire
Zhu Junyan, Ji Fang, Di Wen, Gu Zhuowei	The application of PBL teaching method in the teaching of obstetrics and gynecology	2008	China	Journal of Shanghai Jiatong University	Students	Learning Based on in Problems	No comparison	Greater reasoning clinical Communication skills	Experience descriptive
